# Exploring safety aspects of maternity care through the lens of midwifery students’ clinical experiences in Belgium, Czech republic, Estonia, Norway, Slovakia: A qualitative study

**DOI:** 10.1186/s12912-025-03296-2

**Published:** 2025-07-01

**Authors:** Daniela Javornická, Synnøve Mari Eidsvik Folkvord, Annelies Jaeken, Terézia Krčméryová, Helena Kisvetrová, Mary Steen

**Affiliations:** 1 The Centre for Research and Science, Faculty of Health Sciences, Palacký University Olomouc, Hněvotínská 976/3, Olomouc, 775 15 Czech Republic; 2 Department of Midwifery, Faculty of Health Sciences, Palacký University Olomouc, Hněvotínská 976/3, Olomouc, 775 15 Czech Republic; 3 Faculty of Health Sciences, University of Stavanger, Mail box 8600, Stavanger, 4036 Norway; 4 Healthcare Department, Midwifery Hogeschool PXL, PXL University College of Applied Arts and Sciences, Elfde-Liniestraat 24, Hasselt, B-3500 Belgium; 5 Faculty of Nursing and Professional Health Studies, Slovak Medical University, Limbova 2651/12, Bratislava, 83101 Slovakia; 6 Midwifery in School of Nursing, Faculty of Health Sciences, Curtin University, Kent Street, Bentley, Perth, WA 6102 Australia; 7 DNAMER, King Edward Memorial Hospital, 374 Bagot Road, Subiaco, Perth, WA 6008 Australia

**Keywords:** Safety, Midwifery, Students, Respectful care, Mentorship, Trauma

## Abstract

**Background:**

Meeting the safety needs of women and newborns is crucial in preventing harm in maternity care. Recent recommendations suggest that childbirth needs to be understood through a broader framework, since maternal and newborn mortality/morbidity remain a global challenge. The unique role of midwifery students in clinical environment can provide and contribute to such understanding. This paper explores aspects of maternity care services impacting patient safety as identified by midwifery students in five European countries.

**Methods:**

A 2-stage qualitative design employed an interpretivist approach. Thirty-five midwifery students (Belgium, Czech Republic, Estonia, Norway, Slovakia) were recruited through purposive sampling to engage students with an Erasmus + experience. Stage 1 (2022–2023): Thirty-five written narratives were collected anonymously via an online ‘Sharing LearnIng from Practice for Patient Safety‘ Learning Event Recording Tool. The dataset captured care in antenatal, maternity and postnatal wards. Stage 2 (2024): Three focus group discussions verified and added to the initial findings. Audio recordings were transcribed, NVivo software was utilised to assist reflexive thematic analysis in both stages.

**Results:**

Three themes emerged from the analyses. ‘*Treat me well’ theme* captured the communication gaps and dehumanised assembly-line approach leading to compromised safety, obstetric violence and trauma. The second theme describes the paralysing impact of specific team members in *‘Team dynamics and accountability’*, reducing a sense of agency among staff to advocate for patient safety. Theme *‘Traumatised team-members’* reveals the secondary trauma impact on emotional fatigue and defensive practices.

**Conclusions:**

Midwifery students emphasised the importance of individualised, well-communicated, and respectful care, with language barriers being addressed. Ensuring that patients feel safe seems equally relevant to physical safety. Collaborative teams prevent errors/hazards through interdisciplinary simulations, debriefings and students’ continuous mentoring. By fostering a psychologically safe environment and implementing whistleblowing policies, the paralysing bystander effect among staff might be mitigated, and obstetric mistreatment, violence, and trauma could be addressed. Unresolved secondary trauma appears to increase unnecessary interventions and reduce the emotional availability of staff for patients in maternity care. Hence, effective recovery needs to be supported by management, and emotional resilience training should be incorporated into midwifery curricula to indirectly improve patient safety.

**Clinical trial number:**

Not applicable.

## Background

Multinational policymakers have increasingly highlighted the need for patient safety, as still one in ten patients endures preventable harm by errors during their care [1, 2]. The “Global Action on Patient Safety” led to the development of the Global Patient Safety Action Plan 2021–2030 [2]. In maternity care, the increased awareness of maternal health disparities globally, including countries with high Gross Domestic Product, has led to urgent calls for changes in health care delivery that improve safety and health outcomes for all, irrespective of their origin or socio-economic background [3, 4]. Apart from physical harm in maternity care, which might be easier to monitor, research shows that 1 in 3 births are also experienced as psychologically traumatic. About 4% of women/patients and 1% of their partners develop post-traumatic stress disorder [5–7]. There is evidence that perceived inadequate care during childbirth increases the risk of postpartum depression and suicide in women [8]. Given the context of European countries, recently facing a dramatic decrease in birth rates [9] providing safe and non-traumatic maternity care is a priority to address.

The importance of safety has been widely accepted and explored [10–13]. However, despite the ‘12 Steps to Safe and Respectful MotherBaby–Family Maternity Care’ launched by the International MotherBaby Childbirth Organization (IMBCO) and the International Federation of Gynecology and Obstetrics (FIGO) [14]; despite FIGO’s ‘Childbirth: A Bill of Rights’, aiming to reduce the global maternal mortality ratio to less than 70 per 100,000 live births and to end preventable deaths of children and newborns by 2030 [15], emerging evidence shows a lack of progress towards safety globally [4, 16]. In 2024, the American College of Obstetricians and Gynecologists’ Commitment to Policy Action even refers to ‘the maternal mortality and morbidity crisis’ [17]. Similarly, the findings of the national inspection programme in England by Care Quality Commission, where 48% out of 131 maternity units were found ”inadequate” or “requiring improvements”, call for a robust focus on safety to ensure that poor care and preventable harm do not become normalised and that staff are supported to deliver the high-quality care they want to provide for mothers and babies [18].

Due to slow progress in improvements, recent recommendations call for the involvement of all stakeholders in addressing and seeking a deeper understanding of safety [19]. While health professionals and patients have been traditionally involved in research on patient safety, there is a missed opportunity in not including midwifery students in safety research. The majority of studies have focused more on students’ own clinical learning challenges [20–24]. Therefore, an additional perspective gained through the lens of midwifery students to explore the complexities of safety in maternity care can contribute to gaining insights and improve care for women, their newborns and families. Midwifery students’ experiences provide an opportunity to capture unique patient safety insights. Given that students work alongside health professionals (approximately 50% of midwifery program duration), they experience both exemplary and compromised care, seen through the ‘fresh eyes’ of soon-to-become professionals [23]. Therefore, the aim of this study was to explore aspects of maternity care influencing safety as identified and experienced by midwifery students.

Midwives and midwifery students often practice in complex environments, with the aim to provide gold-standard care. To achieve this aim, they need to work in a healthy environment. There is good evidence showing the relationship between the work environment and patient safety outcomes. For example, high midwife stress seems to lead to more adverse events [25, 26]. Yet, stress is almost inevitable in the midwifery profession per se [27, 28]. Poor working environments combined with frequent staffing concerns can not only increase stress levels and attrition but also the risk of causing harm. Recent data showed that understaffing of registered midwives can be associated with an 11% increase in harmful incidents [29]. Therefore, the estimated shortage of 900,000 midwives worldwide calls for attention in terms of safety [30]. Reducing attrition equals harm prevention and vice versa. Significant relationships exist between the work environment, women’s safety culture, and midwives’ intentions to leave their job or profession [12, 31, 32]. Additionally, it is early-career midwives and midwifery students who tend to question their choice of profession based on their clinical experiences. Their insights and concerns around safety can therefore bring valuable findings, potentially assisting policymakers in clinical and academic settings in shaping recommendations for safer maternity care, internships and workplaces in general. A deeper understanding of the intricate safety matrix seems essential for the well-being of mothers, their newborns and the future midwifery workforce.

## Methods

### Study design

A 2-stage qualitative design employed an interpretivist qualitative approach, as this focuses on how people make sense of events within their social and institutional environments. This approach motivated researchers to seek midwifery students’ experiences and to consider different interpretations of particular social contexts in order to gain the in-depth understanding required [33, 34]. Since patient safety is not only about procedures but also shaped by the context in which care is provided, the interpretivist approach facilitated context-sensitive insights. Findings are presented following the Criteria for Reporting Qualitative Research (COREQ) guidelines [35].

### Participants, recruitment and setting

This study, conducted within academia, explored the experiences of students attending clinical placements in antenatal, maternity, and postnatal wards in their home countries. A purposive sampling strategy was applied to engage students with international Erasmus + experience, allowing for a broader perspective on the research question. Purposive sampling ensured that participants who agreed to participate had been exposed to experiences relevant to the research question in order to obtain rich and detailed information [36, 37]. Erasmus mobility experience ensured not only international exposure, but also English language fluency for the needed ability to express and discuss students’ experiences in written and spoken English.

Inclusion/exclusion criteria were predetermined. Midwifery students with clinical placement experience in any of the clinical areas explored (antenatal, maternity, postnatal) were included. Their exposure to patient safety-related events was mandatory, irrespective of whether or not this was reported as an incident, near-miss or other. Students’ own identification of a situation as ‘safety-related’ met the inclusion criteria. Students without clinical experience in the settings studied and those with incomplete consent to participate in this study were excluded.

Recruitment involved an in-person invitation to students attending an international midwifery summer school at a Czech university, which hosted students from five European countries: Belgium, Czech Republic, Estonia, Norway, and Slovakia. An email invitation provided a link to the study website, along with informed consent and a questionnaire. Participation was voluntary and anonymous via online reflection in stage one, followed by online focus group interviews for those who chose to participate in stage two. All narratives were de-identified before analyses.

### Data collection

Written narratives were collected in stage 1 via completion of the SLIPPS Learning Event Recording Tool online (‘Sharing LearnIng from Practice for Patient Safety‘ Learning Event Recording Tool, SLERT) [38]. This tool was developed to enable students to learn from patient safety events through guided reflective writing. The SLIPPS Learning Event Recording Tool was used to guide students’ reflective writing, as it offers a clear and structured format for capturing experiences related to patient safety. This tool focuses on contributing factors and supports reflection beyond a descriptive account and encourages critical engagement with the event.

In stage 2, three focus group discussions (FGDs) were held with midwifery students who chose to participate in this stage, to verify and enrich initial codes and themes identified in stage 1. The online format of FGDs and the number were selected to enable international students with varying clinical and academic commitments to attend. The FGDs were semi-structured, with prompts based on the reflective accounts, and aimed to create space for students to expand on their experiences, compare views, and explore shared concerns around patient safety. Prompts for FGDs were created by the team based on data analysis from stage one and consultation with two senior researchers on the team.

Questions used in FGDs:

- What would you consider the biggest threat to patients’ safety in maternity care, based on what you have seen on practical placements?
- What do you recall being undertaken around patient safety events in the hospitals you have practised in?

A total of *n* = 35 midwifery students from Belgium, Czech Republic, Estonia, Norway, and Slovakia participated. The participants’ ages ranged from 19 to 35 years. Country-specific curriculum differences are explained in Table 1.

**Table 1:** Midwifery programs overview (countries included in this study)

	Midwifery programs	Duration of the study program	Language	Title
Czech Republic	Bachelor’s Master’s	3 years (direct entry*) 2 years *(optional continuation of Bachelor’s)*	Czech (second language: English as a part of the curriculum)	Bachelor of Science in Midwifery Master of Science in Midwifery
Slovakia	Bachelor’s Master’s	3 years *(direct entry)* 2–3 years *(optional continuation of Bachelor’s)*	Slovak (second language: English as a part of the curriculum)	Bachelor of Science in Midwifery Master of Science in Midwifery
Norway	Master’s	2 years *(prerequisites: Bachelor’s in nursing or other health sciences plus 1-year clinical experience)*	Norwegian (second language: English as a part of the curriculum)	Master of Science in Midwifery
Belgium	Bachelor’s Master’s	3 years *(direct entry)* 2 years *(optional continuation of Bachelor’s)*	Dutch (second language: English as a part of the curriculum, some courses taught in English)	Bachelor of Science in Midwifery Master of Science in Nursing and Midwifery
Estonia	Bachelor’s	4,5 years *(incorporates both nursing and midwifery)*	Estonian (second language: English as a part of the curriculum)	Diploma of Professional Higher Education Bachelor of Science in Health Sciences (BSc)

*Entering a Bachelor program from a non-healthcare-related high school/secondary school

17 students participated in FGDs in stage 2. The students themselves selected their FGD group based on their availability and preferred date and time (Table 2). They received no remuneration for participation. Discussions were audio-recorded and auto-transcribed with participants’ permission via Microsoft Teams. The transcribed data were checked for accuracy. To reduce interviewer bias, focus groups were moderated by a researcher not involved in students’ teaching or assessment. A semi-structured topic guide was used consistently across all discussions. Questions were open-ended and neutrally phrased to avoid leading responses. The moderator maintained a reflexive stance, supported by brief reflective notes after each session.

**Table 2:** Data collection: stages and participants

Participants total: 35 students					
	Stage 1 35 written narratives (M1 – M35)	Stage 2 17 participants (S1-S17)	Focus Group 1 7 students (1 h 13 min)	Focus Group 2 8 students (1 h)	Focus Group 3 2 students (39 min)
Norway	4	5	*		
Belgium	8	2			*
Estonia	7	2	*		
Slovakia	8	3		*	
Czech R.	8	5		*	
Type of program	29 undergraduate midwifery students + 6 postgraduate midwifery students				
Age range	19 - 35				

* Students from these countries participated in that particular focus group discussion

### Data analysis

Reflexive thematic analysis was utilised to gain an in-depth understanding of specific aspects of maternity care relevant to patient safety. Analysis of the written narratives and focus groups’ data utilised Braun and Clarke’s (B&C) 6-phase process [36]. Analytical writing and reflective multi-researcher/multi-national discussions were complemented by NVivo software. Data familiarisation (B&C phase 1) and research-team note-taking were followed by systematic descriptive coding of (B&C phase 2) all narratives in phase 1 independently by three researchers (DJ, SMEF, AJ), findings were compared, and initial semantics, codes and interpretations reflexively discussed amongst the whole research team enabling an opportunity for alternative interpretations. Grouping and regrouping of found categories resulted in the formulation of initial themes (B&C phase 3). Themes’ development and review (B&C phase 4) through analytical discussions (DJ, MS, HK) opened further perspectives. Refining and defining the process (B&C phase 5) resulted in the formulation of themes (B&C phase 6). To support rigour, the process included regular research team meetings and reflective discussions, allowing for reflexive interrogation of interpretations and consideration of alternative perspectives. Consequently, the same data analysis strategy was employed to analyse the data collected in focus group discussions. The third FGD identified no new data being shared. Eventually, data consolidation and enrichment through comparing and contrasting, deepening and clarifying researchers’ insights, assisted in generating final themes. To ensure consistency and reliability, coding was conducted in parallel by multiple researchers in both stages, and findings were regularly reviewed during team meetings. Differences in interpretation were discussed until consensus was reached. To ensure findings were credible and grounded in the data, regular meetings were held to discuss the transpiring findings and complemented by debriefings with senior researchers. Dependability took into consideration feedback from experts, participants, and colleagues with regard to the findings to explain ambiguities and resolve them. Transferability was enhanced through purposive and heterogeneous sampling and expert review comments.

### Ethical considerations

Ethical approval was obtained (UPOL-17700/1070–2020; UPOL-149073/FZV-2023, details below), and participation was voluntary based on informed consent. Participants were aware that their participation would not impact their studies in any way, and they could withdraw from the study at any time. No withdrawals occurred. Confidentiality and anonymity were ensured; narratives and verbatim statements were numbered and kept unidentifiable unless students chose to refer to a specific country in their statements.

### Findings

Three themes emerged from the analysis (Table 3). While the first theme captures the relevance of *‘treating women well’*, impacting their feeling safe and being safe, the second theme encompasses safety aspects influenced by *‘team dynamics and accountability’*. The third theme narrows down to ‘*traumatised team-members’* being viewed as a safety concern, as students identified that unresolved trauma can impact clinical practice and emotional availability for patients and others.

Square brackets/ellipsis are used where words have been added for clarity or removed for brevity (‘M’ refers to written narratives, ‘S’ to interviews).

**Table 3:** From codes to themes

Themes	Subthemes	Codes	Preliminary codes - examples
I. TREAT ME WELL	• Communication barriers • Individual needs lost in an assembly line approach • Obstetric mistreatment, violence and trauma	← Language barrier ← Insufficient provision of information ← Production line: didn’t feel heard or safe to ask, lack of individualised care, lack of control, unconsented care, manipulation, insufficient follow-up ← Obstetric mistreatment and trauma ← Medicalised care and outdated practice	← Language barrier ← Communication, informing patient and family, lack of co-determination rights, informed consent ← „Factory line“, lack of individually adapted care; practitioner-led care, not woman-centred care; task-oriented; lack of compassion, need for respectful maternity care ← Lack of follow-up ← Inhumane behaviour, physical assault, trauma ← Out-of-date practice, gap between theory and practice; clinical practice not based on evidence or physiology
II. TEAM DYNAMICS AND ACCOUNTABILITY	• Unit culture and teamwork • The paralysing power of ‘everybody knows’ • Safe staffing, skill-mix, mentoring	← Interpersonal relationships ← Hierarchy dynamics ← Shortages of staff; under/over experienced ← Debriefing, team drills – leading to a non-blaming culture and good teamwork ← Errors and delays due to a lack of students’ supervision, changing mentors	← Unit culture, miscommunication, and conflict; lack of accountability ← Unprofessional behaviour, unpleasant environment ← Abuse of power, bullying, no action, everybody knows ← Teamwork, communication, debriefing ← Despite hierarchy; wisdom, kindness ← No time for everything, not enough midwives, doing too much for too many; ageing ← Lack of assigned mentoring, no clinical supervision, errors, near-misses, delays ← Not safe to speak up or report, feeling inappropriate to question, no space for discussion; no point in pointing out
III. TRAUMATISED TEAM-MEMBERS		← Secondary traumatisation ← Guilt and self-blame ← Emotional safety	← Helpless ← Devastated ← All my fault ← The staff didn’t ask for help

### Theme I. – Treat me well

#### Communication barriers

Effective communication between all stakeholders in healthcare is a fundamental aspect of patient safety. Midwifery students identified challenges in conveying information to patients/families. Especially in life-saving situations when staff can become overwhelmed by clinical demands or stress, leaving the patients confused, dehumanised and excluded from their own care. Several accounts highlighted that such high-pressure moments limited dialogue and created uncertainty that could compromise safe care delivery and create a risk of mistreatment.

Without any instruction, she was only informed that she was going to have a C-section…She was not instructed about the anaesthesia. And instead of telling the patient that she was going to have a urinary catheter inserted the nurse just said ‘spread your legs’ and gave me the order to catheterize. (M23)

When an emergency CS…everything is too fast. Suddenly too many people… So many things are happening. And the mother and father of that baby don’t actually know what’s happening. So, when we don’t tell them, their fear is growing. (S10)

But you are so focused on saving the baby that you don’t explain to the mom what’s going on. And then she gets scared. (S17)

Providing information is even more challenging due to a language barrier. Students described challenges in caring for women who did not speak the same language, particularly in situations requiring explanation, reassurance, or consent. Several students reflected on moments where women appeared confused or distressed during examinations or procedures, with limited opportunity to ask questions or understand what was happening. The lack of a shared language often prevented midwives, obstetricians and students from providing adequate information or emotional support. One participant highlighted a serious adverse event, a family tragedy discussed on national media, caused by the inability to obtain informed consent due to a language barrier.

The patient couldn’t speak Dutch nor English…was rushed for a caesarean… The mom didn’t understand what was going on …and after several minutes of ignorance she fainted. (M11)

There was a huge incident in our country last year. They took a wrong woman for abortion… They were both Vietnamese. There was no interpreter around. People lost jobs because of that… (S5).

They [woman/family] didn’t speak English at all… They just did what they thought was good for the woman, but they didn’t communicate with her. And I feel like that’s wrong…also these women should know what’s happening. It ended up by CS. And yeah, she didn’t know what they were doing. (S17)

One of the major risk factors in Norway is when we don’t have a translator. For the minority group that doesn’t speak Norwegian or English, that’s one of the biggest risks. (S2)

Students from all included countries highlighted language barrier concerns, resulting in preventable incidents or visible confusion, anxiety, and distress. However, the barriers to effective and empathetic communication seem to be more than just language barriers and urgency.

### Individual needs lost in an assembly line approach

The innate nature of respectful care involves active listening and two-way communication. When individual needs, feelings, rights or dignity are ignored, care becomes dehumanised and task-oriented. Sometimes framed as efficiency by health care professionals, several narratives highlighted concerns that women’s autonomy was undermined in the most intimate and vulnerable moments in their lives.

When it comes to the pushing stage the baby’s heartbeat is already decelerating a bit from time to time, and more and more people come into the room. The situation escalates and her legs are put up in holders, the obstetrician is on standby holding the vacuum, there’s a paediatrician in the room, three midwives, and me, a first year student on her fifth (evening) shift… I was frustrated at the lack of progress the people in the room have made regarding informed consent. I understand that the baby was struggling a bit, but I also know that while doing things or in the split seconds before doing certain things, everyone should have been able to explain or ask for permission. I can appreciate that people can be stressed and forget to explain things, but this to me seemed more like a work place culture that is allowed to go on without questioning. (M5)

If the woman feels welcomed and seen…then she feels safe. But if she is put in a role ‘well, you are just next in line’ because it’s our job-description’…there is no humanity then. (S13)

When the professionals want to do some intervention or treatment…they just kind of like manipulate the woman into agreeing… And she sort of senses that she’s being manipulated and then… she doesn’t know if she can trust those professionals. In that way, she might not feel safe. … Through whether her being healthy is the priority for those medical professionals. (S8)

Often this ‘talking into’/coercion was apparent even in non-acute situations such as choosing a birth position.

...in the end it is ‘my labour and my baby’, despite obstetrician being responsible for it, the woman has a right to co-decide. Like this older doctor – she told him three times she wanted to be on all fours, me and the midwife told him twice. But even before she started pushing she ended up on her back. (S13)

Having the possibility to do interventions when it’s needed… but it is not always needed. I think it’s overdone all over the world…to do something the same way because I’ve been always doing it that way is not good enough reason… (S3).

…then you start working like on a factory band… You start doing the same things and you feel safe in that. You know, you just start treating everyone the same. (S4)

Students were empathetic about women/patient innate need to be in control of their own labour/body and identified that the routinised staff approach led to medicalised care, with an increased rate of interventions which were not indicated by clinical circumstances. These moments were perceived as safety issues in their own right, as they compromised respectful care, consent, and psychological well-being. Students highlighted the need to protect both the clinical and relational dimensions of safety, emphasising that preserving women’s sense of agency is not separate from safe care but an essential part of it.

### Obstetric mistreatment, violence and trauma

Consequently, ineffective communication combined with disrespect or malpractice was viewed by students as a root cause of adverse events, mistreatment/violence and trauma. Instances of inhumane behaviour were distressingly noted by students. Moreover, when it exacerbated an already traumatising event: *[perinatal loss] The main point was the complete lack of communication between the midwives and the woman*,* as they themselves did not know how to communicate. Therefore*,* they visited her even minimally*,* only in necessary cases such as administration of medicines*,* measuring vital signs. (M25)*

[perinatal loss] The medical staff were just being quiet and pretending that nothing was happening and telling the patient that it was going to be okay and to be quieter. They averted their eyes and avoided communication… (M17).

What struck me was that after 10 min [post-delivery] the doctor started to forcefully scrape out the placenta by hand… As a result, the doctor wasn’t sure if the placenta was completely taken out or whether a piece of the placenta was left in her uterus. I felt uncomfortable with this situation. I felt that the doctor had no respect for the patient and was behaving inhumanely. In a way, she could have caused further complications to the patient postnatally. The doctor’s behaviour was compounded by the fact that she was in a hurry to get on a bus to somewhere immediately after the birth. (M21)

The biggest risk is like complications due to trauma… like, emotional, mental related issues. I think lack of control, lack of being seen and heard. Scenarios where they’re not treated with respect or being informed, included… and people are invasively doing things to them without consent. (S4)

It was normal delivery until the midwife asked woman to push at 7–8 cm, after 5 h of pushing, it ended with C.S. At the time woman was pushing, midwife was screaming, that woman is not doing a good work. Later we discussed it and the midwife didn’t know, what went wrong.… (M6).

She had had two episiotomies…and a traumatic experience with giving birth. … Fingers are put inside her vagina by multiple people without anyone saying anything to her beforehand, and an episiotomy is placed without anyone mentioning anything. I almost felt like I was frozen from shock, that this was actually happening [vacuum-assisted delivery]. It was so obvious to me that so much wrong was going on, and everyone seemed to think it was normal and acceptable. …I think with the knowledge we have today regarding the impact of obstetric violence, we should all do better… The significance of informed consent and the empowerment of the birthing woman is huge. (M5)

She [midwife] supervised me and kept telling me to insert the catheter deeper despite the patient’s pain. As I didn’t want to push on due to the patient’s pain, the midwife took the catheter and forcefully inserted it. The result was a traumatized patient and me as well. (M23)

Students often painfully reflected on and highlighted the psychological harm of inadequate support and obstetric violence. Especially when professionals were oblivious of it. Addressing obstetric mistreatment is essential not only for protecting women’s dignity but also for preventing harm. Participants’ accounts suggest that fostering respectful, consent-based communication and creating space for emotional safety during childbirth can reduce the risk of trauma. These findings point to the need for clearer guidelines, students’ emotional support and staff training in trauma-informed care. Supporting students to recognise and speak up about mistreatment may also serve as a pathway to long-term cultural change in clinical practice.

### Theme II. – Team dynamics and accountability

While the first theme encompasses aspects of safety related to communication towards patients/families, respecting individual needs as opposed to routines, the second theme describes underlying internal factors promoting/inhibiting safe care stemming from unit cultures, team dynamics or unavailable human resources.

### The paralysing power of ‘everybody knows’

A disturbing safety-inhibiting factor seemed related to specific personality traits in many narratives. As if a domineering behaviour of certain individuals prevented other professionals from acting against misconduct and/or malpractice. As if the ‘well-known/accepted aura’ or fear around such colleagues paralysed accountability and sense of agency.

One doctor here, she is perhaps well known everywhere… nobody tells her anything as she does it her way anyway. …labouring women protest against her but she refuses to leave the room. And the women can’t do anything, they can’t stop giving birth, right… (S13).

Since this moody senior doctor, who everyone is afraid of, came in, things started to go wrong. The nurse suddenly didn’t even know which end (of tubing) belonged to what, how to connect it, then she disconnected the long set, but before she stopped the transfusion there was blood everywhere… (M28).

One physician…he has this thing with birth plans… This doctor does episiotomy to every woman, primi…multips… he just always does it. And if the woman totally refuses it then he purposefully doesn’t protect the perineum at all. So that she gets a perineal tear. And the staff know… (S12).

The head of neonatologists has a deep-rooted old NRP algorithm. He is aware of the new one, but is convinced his routine is the best. …like this planned CS, baby was well and he insisted on deep suction… he basically caused the vagal reflex and cardiac arrest just because of his ego…he takes it as a confirmation how great doctor he is. He rescued the child. (S8)

In several accounts, students observed that certain individuals—often more senior or long-established in the unit—were perceived as untouchable, even when their actions conflicted with evidence-based recommendations and respectful care. This unspoken acceptance of misconduct, possibly rooted in fear or resignation, discourages others from speaking up. Such a climate of silence can increase the likelihood of avoidable harm.

And even if there is a proactive staff member advocating for patient, the ignorance of the perpetrator might prevent future attempts when not supported by the whole team:

…the patient pointed out the fact that she could feel her limbs. The surgery began and the patient’s crying and screaming echoed through the OR, the doctor [obstetrician] asked for general anaesthesia… but the anaesthesiologist did not respond… The anesthesiologist who administered the anesthesia is known for his personality and lack of empathy for others. (M29)

### Unit culture & team-work

When able to compare, students could see differences between good versus non-healthy teams and the impact on safe clinical practice. The importance of teamwork, despite hierarchy, for the benefit of a good outcome was highlighted.

[shoulder dystocia] The gynaecologist had frozen after doing the manoeuvres …the midwife had to take over. …the gynaecologists are seen so much higher than the midwives here… It made me feel more thankful of my job. … she was so thankful we were there because she didn‘t know what happened. (M15)

The promptness of team response and respectful team-spirit were admired by students as something to work toward when providing safe care.

I saw vacuum extraction and the collaboration between those doctors and midwives was on a whole different level…the communication… they’re equal here… it’s so nice to see. (S9)

I’m in a respectful environment, there’s collaboration between midwives and doctors… they trust each other, they communicate together like…“I’ll just give you some more time to try some of the alternative things before we move on to some interventions.”…that’s a big shift. (S10)

Open communication and mutual respect supported quicker decision-making and better coordination of care. In contrast, poor teamwork was associated with delays, misunderstandings, and a lack of trust, all of which could pose direct safety hazards, especially in time-sensitive or high-risk situations common in maternity care.

The atmosphere on the unit - it is sometimes obvious there is something going on between the staff… when there are some frictions they radiate it around. (S13)

In my opinion, this was preceded by long-term disagreements. Upon arriving in the room, the midwife asked the nurse to help her with the patient [transfer postpartum by wheelchair]. The nurse objected that she was not going to help because she had a backache and that she should do it herself, having brought the patient in poor condition… As the patient stood up, after about 20 s, the patient suddenly turned pale and fainted…. The nurse at that point started screaming at the midwife that it was her fault, to which she screamed back that she should get out. This situation resonated with me because I couldn’t imagine how bad staff relationships could be. And how directly it almost damaged the patient’s health. (M16)

A lack of multidisciplinary debriefings seems to lead to a polarised workplace and blaming culture.

It would be good when something like that happens [acute events], if more people from that team would just get together, which I’ve hardly ever encountered. It’s more like maybe the midwives just discussed it with each other in the nurses’ station…but in most cases, doctors weren’t really involved in it, it’s just a separate world…. (S8)

…the blame was thrown on the midwives. (S9)

I think here is closer contact with the doctor, back home it isn’t. They [midwives] have to listen to the doctor. You can’t say anything… you do what the doctor says. (S16)

The aspect of midwifery students being part of the team and prepared to provide safe care was highlighted in students’ reflections. They identified not being involved in simulations/drills within their placements or poor orientation to the unit as a safety risk.

Actually, just the fact that we don’t know where the different tools or medications are in that workplace…and then there’s like an increased sense of just helplessness or incompetence when an acute event happens. Maybe it could have been better if we had just practised ahead of time in that workplace. To say‚ OK, we’re just going to try out the situation now. (S8)

Meanwhile in some hospitals and universities, drills/simulations have been already integrated.

This is actually exactly what is being done in Norway here with students in the hospital… I think it’s great because they take away a lot from it… (S9).

In Estonia we have very good simulation classes, we deliver babies or have different situations at school. And it helped me a lot.…because we are going to make mistakes. (M3)

### Safe staffing, skill-mix, mentoring

Yet good teamwork also requires appropriate resources. Our data show that shortages of staff, inadequate skill-mix or absence of a familiar mentor can lead to delays in attending to patients’ needs, missed care or to unnecessary interventions, offered just to gain spare time to provide care to other patients.

They don’t even really ask the patients. They are more like ‘almost everyone takes an epidural’. It’s more like a standard thing, yeah.…midwives have to do a lot of things for a lot of patients. …the midwives don’t have the time to be with the women. When they don’t have an epidural, yeah, the women need us more and we can’t really do that in Belgium. (…) I’ve seen only one birth without epidural. (S17)

Yeah, there’s not enough staff for every task and for the patients. It is not every shift, but it happens. Maybe someone has to wait for a longer time. (S2)

There isn’t a system that automatically picks up on every woman. It’s more if they had a complication or Caesarean. But not after the normal births. We try to have the postpartum appointments or conversations. But there often isn’t enough time to do it. (S2)

The link between staffing and safety clearly emerged. This factor also impacts the actual availability of midwives to fully support midwifery students on placements. Data suggest that students are sometimes left alone or with junior midwives. This lack of guidance poses risks to patient safety. Lack of supervision led several students to near-misses/errors, particularly related to medication administration (for which they were then blamed and even bullied) or to timely provided care. Students were more likely to share their medication error incidents in written narratives.

…my patient was allergic to Penicillin… [mentor] came into the room where I had already gone to attach the lady’s IV set… She screamed in horror to stop…the mixed Penicillin wasn’t marked on the bottle and I injected Clindamycin into that same bottle, but my classmate [who prepared the Penicillin] didn’t say anything. (M18)

[Lower dose of Gynipral commenced] My mentor told me to prepare the Gynipral infusion and she run off somewhere in the meantime…After about 20 min, the screams of another mentor I used to work with earlier (fear and terror) were heard. (M19)

I didn’t notice that the lady had a GDM [gestational diabetes] and so I prepared 2units of oxytocin in a 500 ml 5% Dextrose solution… The mentor had to run to another delivery room and instructed me to go and administer the infusion. (M24)

Very specific aspect emerged when students reflected on their experience working with another than their assigned mentor, reaffirming the safety benefits of continuous mentorship. Through a sense of trust built over time, a good mentor-mentee collaboration can not only enhance learning but can also directly impact patient safety:

My mentor was sleeping and I had a temporary one [official nap-time in some countries]. …I told her we have decelerations, we have very thick meconium…. and she told me “yeah, it was green before”. …it took too much time for her to actually come to the room…night shifts aren’t the safest time to be there like a student midwife… I believe if I had been with my own mentor…it would have taken me maybe like one second to inform her “come in - we need to do something now”. (S1)

It was like not my usual midwife because she was ill, so I had another one that I’ve never been with. …Then the lady was bleeding quite a bit and I said I would like to weigh this. …I felt like it’s a bit too much. That midwife said “no, it’s within the limit, it’s fine”. [eventually PPH, total loss 3600 ml] …very frustrating. (S4)

### Theme III. – Traumatised team-members

Challenging situations, adverse outcomes and other potentially traumatising occurrences are part of midwifery. Students brought up the aftermath of such events in terms of the vulnerability of staff/students, noticing also the impact of such experiences on professionals’ behaviour and on informing clinical practice.

[perinatal loss] I was in a state of shock and disbelief, struggling to process the emotional impact of the situation. I noticed that my supervisor was also deeply affected… It made me acutely aware of the emotional toll that healthcare professionals may experience when dealing with adverse outcomes and the importance of seeking support and practising self-care. (M7)

[Ineffective spinal anaesthesia at CS] A beautiful moment turned into a nightmare that was horrible for both, the lady and the staff who witnessed the moment. (M29)

…they were grumpy and tired of their own work, and then radiated negativity. There was emotional exhaustion… the behavior and mood of nurses/midwives directly affects patients. (M8)

Eventually, the woman gave birth to a dead child, but I was able to support and guide the couple in this. Like taking a footprint, a bath, putting on clothes…. The midwife asked me if everything was okay… But the rest [of the team] no one asked them… I learned how important debriefing is, and how important communication is in workplace. During the event I was little shocked, this was my first delivery of a stillborn child. Afterwards, I was able to give it a place. I learned that I should always ask for a briefing even if I was busy with something else. (M14)

And then you want to put internal monitoring on everyone… just because you feel safe. (S4)

When secondary trauma is addressed and recovery supported, such experiences can eventually become part of professional/personal growth. On the contrary, certain routines and clinical decisions of some professionals can have an emotional root cause. An unresolved secondary trauma might lead to defensive practice, post-traumatic stress symptoms and/or burnout.

## Discussion

This study contributes to a broader understanding of patient safety by the future generation of the midwifery workforce. The themes drawn from our data confirmed the multifaceted nature of safety phenomena in maternity care in the European context [39, 40]. Our findings highlight the interplay between external influences (unit culture, teamwork, staffing, resources, mentorship, psychological safety at the workplace, recovery support) and intrapersonal characteristics (personality traits, emotional resilience, sense of agency, and accountability) that impact patient safety attitudes among maternity staff.

Students observed that providing care in antenatal, maternity, and postnatal areas might often become task-oriented, lacking compassion and respect. Other concerns stem from teamwork-related issues and unresolved second victim trauma. One concern leading to ignored obsolete practices, mistreatment or obstetric violence getting unaddressed; the other resulting in emotional fatigue and defensive practices.

Routinised care, which overlooked individual needs, led students to question whether safety was always prioritised over efficiency. A fast-paced approach led to non-individualised and at times, unexplained and unconsented care, causing distress or trauma. Many studies show that language barriers and ineffective communication pose significant risks to patient safety. These issues hinder the ability to obtain informed consent, can lead to psychological distress, critical errors, and/or underutilised healthcare services [41, 42]. Lack of clear and respectful communication within the healthcare team is one of the most common root causes of reported maternal and perinatal sentinel events [43]. A large study conducted among 3429 labour and delivery nurses in 253 hospitals in the United States linked missed care in maternity units to poor communication and teamwork [44]. In our study, ineffective communication, irrespective of language, was mainly associated with acute events and perinatal loss. There is sufficient evidence that human connection and communication are especially important during high-risk and emotionally uncomfortable moments of care, since they present a potential risk for both physical and emotional safety [18, 44, 45]. Multidisciplinary teams should therefore focus on good teamwork, as well as on the provision of emotional support during rapidly changing situations to mitigate the potential for physical and emotional harm. Efficiency should not be the only measure of quality care.

The dehumanising impact of uncommunicated and routinised care, where patients become invisible, passive participants in their own care, is well captured by Keedle et al. (2024) [46]. 626 patients’ narratives reported bullying, coercion, non-empathic care, and even physical and sexual assault. Disrespect, abuse and non-consented vaginal examinations were the most frequent subcategories. Our data captured similar occurrences. In a large European study (*n* = 27173), women who perceived their care as less respectful reported a higher number of medicalised interventions [47]. This aligns with our findings, as students noted the tendency of staff to intervene without clinical justification. However, they also highlighted the reluctance of staff to intervene when concerns were escalated by students only, undermining their clinical judgement and trust. Interventions can prevent both perinatal morbidity and mortality. However, the medicalisation of otherwise normal maternity care by using unnecessary interventions has negative consequences and has the potential to harm women, physically and mentally, and their newborns [43, 48].

Students’ narratives emphasised concerns arising from observed obstetric mistreatment and violence, interwoven throughout all three themes. Staff displaying such behaviour, as well as team-members ignoring or disregarding it, presented a significant challenge not only to women but also to students and other team-members. Such situations caused direct physical and psychological harm (incorrect neonatal resuscitation, unconsented episiotomies, undergoing CS with ineffective anaesthesia. Mistreatment, lack of control and adverse events can lead to profound trauma in all stakeholders; as well as to emotional fatigue, secondary traumatic stress and burnout in staff [5, 7, 49, 50]. Despite the large body of research addressing obstetric violence, this phenomenon still seems to escape eradication globally [46, 49–51]. Even when terminology is adjusted to distinguish violence from mistreatment [52], there needs to be more exploration of the reasons why institutions are unable to prevent such occurrences. In some settings, midwives are expected to defer to doctors, limiting their ability to advocate for patients effectively. Additionally, having domineering personalities on teams, especially if higher in the hierarchy, can have a detrimental impact on patients, preventing fast and appropriate team action [16, 53, 54]. The power of ‘everybody knows’ presents a new concern contributing to the slow progress in addressing mistreatment and violence.

Low psychological and emotional safety in units can lead to reluctance to voice concerns and advocate for patients [53]. Such a work environment could further normalise the bystander behaviour in teams [55–57]. This concept describes that the more people are involved in a critical situation, the less likely someone is to act on adversities. Diffusion of responsibility, fear of being judged by others when acting publicly or pluralistic ignorance. A maximum bystander effect occurs when nobody intervenes because everyone believes that no one else perceives an emergency [56]. Even our narratives suggest that some teams are blind to an obvious concern, leaving students shocked and traumatised. Even though the bystander phenomenon diminishes in dangerous emergencies [55], in less escalated circumstances, staff appear paralysed by the complex psychological and hierarchical interplay and poor psychological safety.

Conversely, in highly collaborative environments, teamwork and mutual respect among healthcare providers seem to lead to better outcomes [40, 58]. To improve teamwork and outcomes, obstetric simulations have been implemented locally and nationally in many countries. Research confirms that in situ, multi-professional simulations show the best outcomes [58, 59]. Consistent with these studies, our data show that midwifery students appreciated a workplace where care was discussed in multidisciplinary teams and cases were debriefed. Students also expressed an interest in participating in clinical simulations to feel included, competent and prepared for emergencies; however, sufficient staffing and managerial support are required to implement this approach more widely [20, 22, 29]. Bryant and Greenawalt (2024) reaffirm the need to utilise the modality of simulation in midwifery academic settings as a method for reducing errors and increasing patients’ safety in the maternity setting [59].

**Fig. 1:**
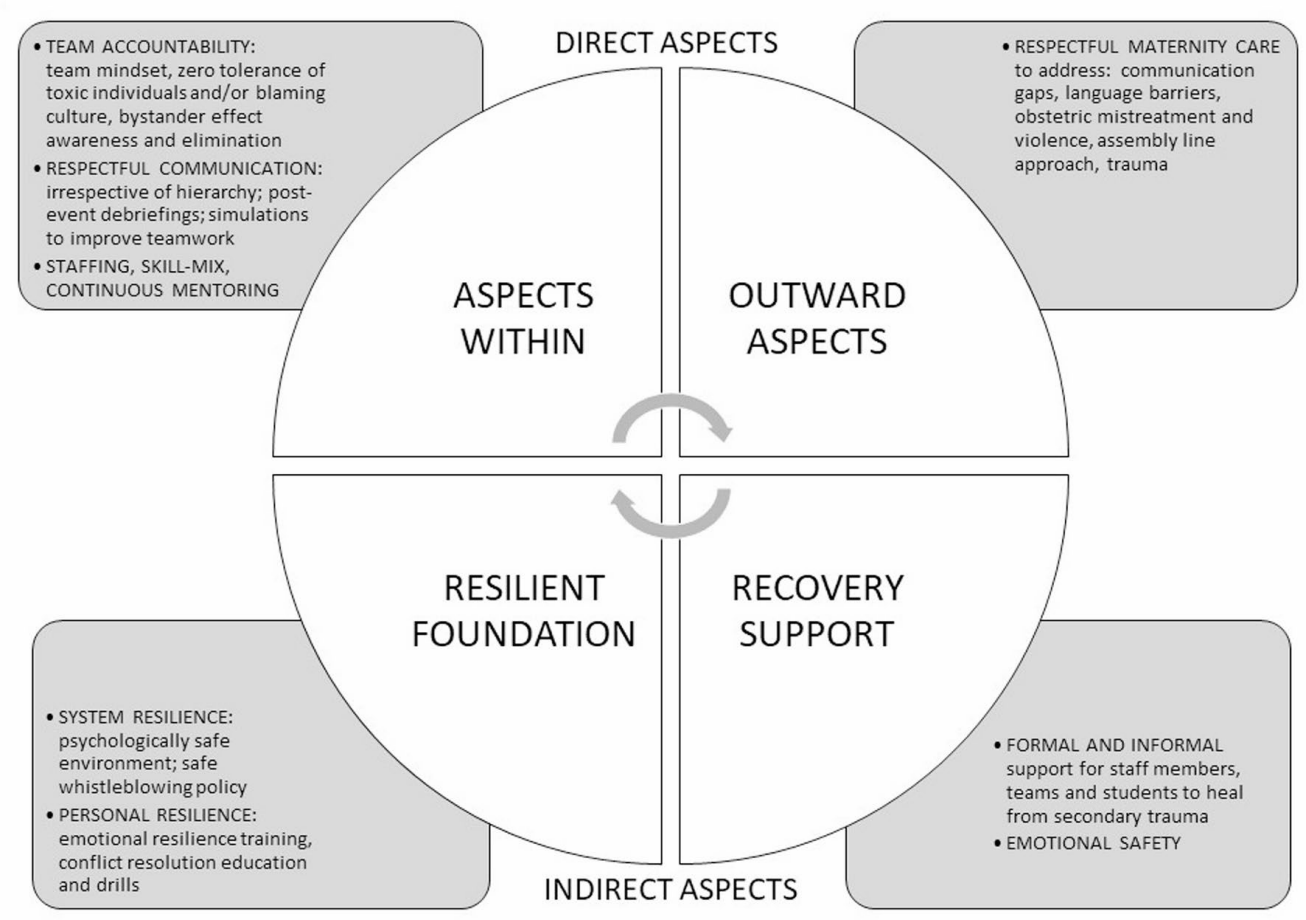
Patient Safety Matrix Foundation (PSMF) summarises recommended approaches to address specific patient safety concerns. To establish a firm foundation for safe maternity care, all four dimensions of safety aspects should be carefully evaluated and addressed

To display the recommendations addressing the emerging aspects of patient safety in maternity care, a Patient Safety Matrix Foundation is proposed (PSMF, Fig. 1). Due to the underlying complexity, there needs to be a multifaceted strategy developed and tailored to the needs of each clinical setting, addressing their unique challenges.

- The need for respectful maternity care (RMC) in ensuring patient safety has emerged [3, 60, 61]. Cantor et al. (2024) in the recent systematic review provide an overview of concepts related to respectful maternity care and 12 validated tools to measure RMC. Framework themes correlated with aspects identified in our study: abuse, consent, privacy, dignity, communication, safety, and justice [3]. Cantor et al. recommended further research exploring specific outcomes of implementing RMC on maternal and newborn safety.
- To improve communication and collaboration, mandatory interprofessional meetings, acceptance of subjective mistakes, mutual understanding, and debriefings of acute events and conflict situations are recommended. Emergency trainings, trainings in effective communication, and handovers are proposed to reduce the risks of preventable adverse events [58, 62–65]. Sansregret et al. (2023) highlight that simulation is most effective when it involves interprofessional collaboration, institutional support, and regular repetition [58].
- Promoting safer working practices can be accomplished by management supporting psychological safety. Further research is needed to verify the effectiveness of interventions mitigating the bystander effect among multidisciplinary teams. A broad synthesis by Capper et al. (2024), exploring the reality of whistleblowing in maternity and newborn settings, points to a lack of primary studies. While whistleblowing can expose poor practices and disrupt power dynamics, open disclosure requires psychological safety [66].
- Recovery from secondary trauma seems to be no longer only a matter of self-care. Research suggests that nursing and midwifery managers, as well as academia, should help to improve the emotional and psychological resilience of midwives and midwifery students to reduce individual stress perception, enhance coping abilities and achieve post-traumatic growth [67–71].
- The inclusion of conflict resolution and resilience-building education within midwifery curricula may equip midwifery students with the skills to manage and address conflict in the workplace [72]. Incorporating emotional resilience into midwifery programs can ensure a supportive workplace environment for patients and staff, preventing burnout and reducing attrition in future [72–74].
- Many narratives in this study are congruent with the evidence that poor rapport between midwifery students and the mentors/teams, as well as staffing constraints, can lead to medication errors. Additionally, a genuine clinical concern can then be missed and ignored, despite students’ escalation. Cumulative evidence shows a causal relationship between the lack of students’ supervision and errors/near-misses [20, 22, 29]. Supportive and supported mentoring can prevent safety hazards.

Unlike previous studies that mainly explored safety through incident reports and adverse outcomes, our findings uncovered more intricate aspects that co-create the foundation of safety. This study adds to the growing body of evidence highlighting that patients in maternity care want to be safe, but their “feeling safe” matters, too [75].

### Strengths, limitations and implications

The strength of this study was the availability of an international team for the analysis, together with senior researchers’ support. To our knowledge, research with midwifery students has primarily focused on general phenomenological grounds, whereas core safety research has involved professionals, patients, and leaders, but overlooked the observational capacity of students. This study aimed to utilise this unique capacity to gain fresh perspectives.

The study has limitations as the number of participants can not represent overall international aspects, nor country-specific generalisations. Our data show that practices and unit cultures vary even from hospital to hospital within each country. The goal of this study was not to compare differences between countries but rather to find commonalities in safety aspects despite cultural, academic and geographical specifics.

Presented findings suggest improved safety could be achieved through (1) implementing respectful maternity care, (2) addressing language barriers and ineffective communication, (3) ensuring a psychologically safe environment allowing safe and effective whistleblowing. (4) Good teamwork can be achieved by conducting regular multidisciplinary simulations/debriefings and allowing midwifery students to participate. Clinical management should provide strategies to support (5) second-victims’ recovery, to ensure resilience and reduce defensive practice. Academia should closely cooperate with clinical management to (6) establish good rapport around mentoring, as supported and supportive continuous mentoring seems to best prevent errors/near-misses. Further research is needed to explore the bystander phenomenon with respect to addressing obstetric mistreatment and violence, affecting individual accountability among multidisciplinary maternity teams.

## Conclusions

Our data display a complex interplay of factors influencing patient safety in maternity care from the perspective of midwifery students. Effective communication, respectful maternity care principles, psychological safety with supportive unit culture and teamwork are critical components of safe maternity care in all five countries involved in this study. Whilst the level of medicalisation in maternity care and inequality within multidisciplinary teams differed from country to country, these variations were also apparent from hospital to hospital within each country. Hospital policies should be updated to address language barriers, obstetric violence and trauma, and to facilitate whistleblowing and the safeguarding of patients, staff, and students. Close cooperation between academia and hospitals can ensure the support of mentors in continuous mentoring and the involvement of midwifery students in clinical teams’ simulations, as both strategies are essential to prevent harm. The inclusion of respectful maternity care principles, resilience-building strategies, effective communication training, including conflict resolution, within midwifery curricula may equip midwifery students with the needed skillset, ideally prior to clinical placements.

Moreover, supporting emotional resilience and secondary trauma recovery is crucial for sustainable improvements in maternity care. Incorporating emotional support and training into hospital policies can minimise defensive practices, emotional exhaustion among staff, and reduce unnecessary attrition. Strategies to address the paralysing bystander effect by enhancing individual and team accountability, preventing the exposure of women/patients/staff to mistreatment, trauma or physical harm, require further research.

By addressing mistreatment, unit culture and teams’ performance, healthcare systems can work towards ensuring safer, more compassionate and respectful maternity care. Being ‘respectful’ has long been more than a ‘nice-to-have’ aspect of care, but a recommended standard. The shift of paradigm away from prioritising physical safety to considering also psychological and emotional safety when caring for patients is well-recognised by midwifery students.

## Data Availability

The data that support the findings of this study are not openly available due to reasons of sensitivity and are available from the corresponding author upon reasonable request.

## Abbreviations

CS / C-section: Caesarean section
COREQ: Consolidated criteria for reporting qualitative research
FGD: Focus group discussion
NRP: Neonatal resuscitation program
OR: Operating room
PPH: Postpartum haemorrhage
PSMF: Patient Safety Matrix Foundation
RMC: Respectful maternity care
SLERT: SLIPPS Recording Tool
SLIPPS: Sharing LearnIng from Practice for Patient Safety
WHO: World Health Organisation
